# Changes in Physical Activity Behaviour and Psychosocial Correlates Unique to the Transition from Primary to Secondary Schooling in Adolescent Females: A Longitudinal Cohort Study

**DOI:** 10.3390/ijerph16244959

**Published:** 2019-12-06

**Authors:** Kate Ridley, James Dollman

**Affiliations:** 1 College of Education, Psychology and Social Work, Sport, Health, Activity, Performance and Exercise (SHAPE) Research Centre, Flinders University, Adelaide, SA 5042, Australia; 2 Division of Health Sciences, Alliance for Research in Exercise, Nutrition and Activity (ARENA), University of South Australia, Adelaide, SA 5001, Australia; jim.dollman@unisa.edu.au

**Keywords:** children, adolescence, sedentary, school, recess, girls

## Abstract

Few studies have investigated physical activity changes over the transition from primary to secondary school. This study assessed change in physical activity and the psychosocial correlates across 12 months in two cohorts of adolescent girls, who were either in primary school in year 1, moving to secondary school in year 2 (Transition cohort) or an older cohort (Secondary cohort) who remained in early secondary school. Female adolescents (*n* = 191; 38% response rate) in South Australia self-reported physical activity and psychosocial correlates of physical activity. Changes between baseline and 12-month follow-up were assessed using paired t-tests. Multiple regression modelling identified psychosocial predictors of physical activity change. Physical activity declined in the transition but not the secondary cohort. The decline was most pronounced during school break times. Independent predictors of physical activity change were: change in enjoyment in the transition cohort; and changes in enjoyment, perceived outcomes, and friend encouragement in the secondary cohort. Transitioning from primary to secondary school is a critical period during which physical activity typically declines, particularly among females. Effective physical activity promotion in this vulnerable group will depend on a deeper understanding of the sociocultural, curricular and environmental influences on physical activity that are unique to each school context.

## 1. Introduction

Steep declines in physical activity have been reported among children as they transition to adolescence, and females are at particular risk. In addition to a decrease in total physical activity, participation in vigorous physical activity shows a marked decrease, while time spent in screen-based sedentary behaviours increases during adolescence [1,2]. These changes in intensity of activity are particularly concerning as emerging evidence suggests vigorous activity has specific beneficial effects on cardiovascular disease risk [3] and metabolic health [4]. Conversely, increased screen use in adolescents has been associated with unfavourable weight status, sleep problems, musculoskeletal pain, and depression [5,6].

Numerous studies have reported declines in physical activity and increases in sedentary behaviour within and out of school hours during this time of transition [1,7,8,9,10]. These behaviour changes appear to be most marked when children move from primary (elementary) to secondary levels of schooling [10]. An Australian longitudinal observational cohort study [9] tracked students transitioning from primary to secondary schooling to compare change in physical activity and sedentary behaviour between students who remained at the same school (i.e., schools that encompass primary and secondary schooling) to those who moved schools (i.e., from a primary-only to a secondary-only school). The study found that while both cohorts’ overall physical activity declined over the five- to eight-month period, the declines were more pronounced for those who moved schools, particularly during lunch and recess periods. The behaviour changes were also not restricted to the school day, with students who moved schools also reporting significantly increased leisure-time screen use [9].

The underlying causes for these observed changes in physical activity behaviour are complex. The social-ecological model of health behaviour helps us better understand physical behaviour change by categorising factors that influence physical activity into dimensions of the individual, social environment, physical environment, and policy [11]. When students transition from primary school to secondary school, changes occur across all dimensions of the social-ecological model. Some age-related changes are individual in nature, such as shifting attitudes, activity preferences, and self-efficacy [12,13]; while others are likely to be socially driven, such as changes in social norms, parent, and friend support. These age-related changes in correlates of physical activity occur even without a change in school. However, moving school results in additional changes in factors known to influence physical activity. Social changes that occur during adolescence are compounded by a likely change in peer group and differences in school culture [14]. Built physical environments and school physical activity policies can also differ between primary and secondary schools. The SPEEDY study in the UK [15] found that despite secondary schools reporting a higher number of medium to high-quality sport facilities and greater provision of lunchtime extra-curricular physical activity provisions, the duration of school-break times was significantly less than in primary schools. Secondary schools were also less likely to have compulsory outside activity time during dry weather and were more likely to allow screen-based activities during break time. Notably, of these school environment factors that differed across schools, only those more prevalent in primary schools, i.e., longer duration of break time, compulsory outdoor breaks, and break time activity rules, were associated with greater accumulation of total daily physical activity [15].

A deeper understanding of the determinants of change during the transition from primary to secondary schooling will better enable schools, teachers and parents to support girls’ physical activity. There is a plethora of physical activity correlational evidence for this age group, but it is largely restricted to cross-sectional survey data [16]. While these study designs are useful for explaining pre-existing activity patterns, they tell us little about how these factors need to change in order to change physical activity. More longitudinal study designs, examining the change in both physical activity behaviour and correlates, are necessary. The aim of this study was to assess changes in self-reported physical activity behaviour and psychosocial correlates of physical activity in two cohorts of adolescent girls over one year. One cohort transitioned from the final year of primary school to the first year of secondary school, while a parallel cohort moved from the first year to second year of secondary school. This design allowed us to identify factors that could be incorporated into interventions that are unique to the transition period as well as strategies to maintain regular physical activity in girls while in secondary schools. Specifically, we hypothesised that the transition period represents a significant period of adjustment for young girls and that unique associations of physical activity levels with psychological and social factors would be identified in this group. 

## 2. Materials and Methods

The study was a two-group cohort design, with measurement points in 2011 and 2012, spanning the years from grade 7 (final year of elementary school) to grade 9 (second year of secondary school). One cohort (‘Transition’) was followed from grade 7 to grade 8 and another (‘Secondary school’) from grade 8 to 9. 

### 2.1. Recruitment and Maintenance

A priori sample size calculations were based on an estimated 5 independent predictors of the outcome variable per domain (psychological and social) per cohort in multiple regression modelling, and the power to detect a model R^2^ of 0.20 at a power of α = 0.05 [17]. A total sample of ~200 was targeted in Year 1 to account for a conservative 50% retention from year 1 to year 2, resulting in *n* = 50 in each cohort who provided full data at both time points. In 2011, nine grade 7 students per primary school (‘Transition’) and nine grade 8 students per secondary school (‘Secondary school’) were randomly sampled, with replacement, from enrolment lists in 12 primary schools and 12 secondary schools in metropolitan Adelaide. These schools were proportionally selected from tertiles of the school-card register (SCR: a school ranking system representing the proportion of students receiving means-tested government assistance to meet the cost of school attendance) across all eligible schools in metropolitan Adelaide. Accordingly, sampling represented the range of socioeconomic status in the eligible population. Sample maintenance strategies were implemented, including birthday cards, project updates, and reminder letters prior to follow-up assessment between 2011 and 2012. 

### 2.2. Measures

#### 2.2.1. Maturation

Biological maturation was assessed with the Pubertal Development Scale (PDS), a self-report instrument with acceptable reliability and validity [18]. Five physical changes in pubertal development are scored on a four-point scale, with an overall pubertal status score represented by the mean of the 5 items. Children reported their date of birth at time point 1 and decimalised age was calculated.

#### 2.2.2. Physical Activity Measurement

Habitual physical activity was assessed using the Physical Activity Questionnaire for Older Children (PAQ-C), a validated 7-day recall tool for use with children and adolescents [19]. The PAQ-C incorporates the following physical activity contexts: physical education (PE); school break periods (recess and lunch); and home-based physical activity (right after school, on school evenings, and on the last weekend). A composite index is calculated from the average of seven items to reflect the overall physical activity level (PAQ score; range, 1–5). The PAQ-C has been used in many countries and has acceptable validity and reliability in the age range of the current study [19].

#### 2.2.3. Child-Reported Psychosocial Correlates of Physical Activity

Items in the children’s questionnaire used to derive the psychosocial correlate variables were taken in full from the ‘eat well be active’ (ewba) Community Programs conducted in metropolitan and rural South Australia between 2006 and 2009 [20]. The ewba questionnaire was derived from items on personal attributes and parental support in the Children’s Physical Activity Correlates (CPAC) Scale [21] and items on peer influence in the New South Wales Schools Physical Activity and Nutrition Survey [22]. Finally, a question was created on the influence of bullying on children’s physical activity, as a contemporaneous study in Australian elementary schools identified bullying as a deterrent for physical activity in some children [23]. While psychometric properties of the questionnaire in the current study are not available, a study investigating the predictive utility of the CPAC Scale, from which the majority of items were derived, reported good reliability and demonstrated that 15–20% of the variance in PAQ-C scores was explained by personal and parental influences in middle and high school students in the USA [24].

#### 2.2.4. Personal Correlates of Physical Activity

For all of the measured personal variables, the response options were: strongly agree, agree, unsure, disagree, and strongly disagree. Perceived outcomes of regular physical activity were assessed with the following, beginning with ‘Playing games or sports over the next year might help me...’ and ending with: ‘keep me healthy’; ‘get me fit or help me stay fit’; ‘study and learn better’; ‘have lots of fun’; ‘make my parents/carers happy’; ‘spend time with my friends’; and ‘make new friends’. A ‘Perceived Outcomes’ factor was derived as the average of individual outcomes (Cronbach alpha = 0.77). Barriers self-efficacy was scored with items beginning with ‘I could still play sport or games even if...’ and ending with ‘others made fun of me’, ‘there was no-one to do it with’, ‘I was not good at it’, ‘I had no help to get to training and games’, ‘my parents/carers did not encourage me’, and ‘my friends did not take part’. A ‘Barriers self-efficacy’ factor was derived as the average of individual outcomes, with a Cronbach alpha of 0.78. Single items that did not form factors with acceptable internal structure represented: enjoyment of physical activity (PA), ‘I like playing sports and games’ (‘Like PA’); perceived competency, ‘I think I am good at sports and games’ (‘Good at PA’); and personal barriers, ‘I don’t like how being active makes me feel (e.g., hot, sweaty, out of breath)’ (‘Don’t like PA feel’).

#### 2.2.5. Interpersonal (Social) Correlates of Physical Activity

‘Parent support’ was calculated as the average of four items, with a Cronbach alpha of 0.71; ‘How often does your father/male carer (or mother/female carer) help you to play some sort of sport or physical activity, for instance, take you to sport or give you money for sport?’; and ‘How often does your father/male carer (or mother/female carer) encourage you to play some sort of sport or physical activity?’. The following items on parent influence were entered into models separately, as the Cronbach alpha was substantially reduced when added to the previous two parent support items: ‘How often does your father/male carer (or mother/female carer) play some sort of sport or physical activity with you?’ (‘Parent play with’); and ‘I have the right clothes or shoes for sport’ (‘Clothing’). Rules imposed by parents were represented by a single item: ‘My parents/carers let me watch as much TV as I like at home’ (‘Rules’).

Positive influence of peers was represented by the following single items: ‘How often does your best friend or their family encourage you to play some sort of sport or physical activity?’ (‘Friend encourage’); and ‘How often does your best friend play some sort of sport or physical activity with you?’ (‘Friend play with’). Barriers associated with peers were represented by the following single items: ‘Other kids make fun of me when I am physically active’ (‘Other kids tease’), and ‘It is not safe to play at school because of bullies’ (‘Bullies’). For all of the social variables, response options were: strongly agree, agree, unsure; disagree, and strongly disagree.

### 2.3. Statistical Analyses

Where required, predictor variables (factors and single items) were re-coded so that higher scores could be hypothesised to predict a higher PAQ-C score, for ease of interpretation of results. Descriptive statistics are presented as mean (SD) or category percentages. Changes in PAQ-C score between baseline and 12-month follow-up were assessed using paired t-tests separately by cohort. Changes in the identifiable contextual components of the PAQ-C score (school PE, school lunch time, after school; evening and weekends) were statistically tested using the Wilcoxon signed-rank statistic for non-parametric data. Changes in predictor variables between time points were also tested using paired t-tests for factor scores and the Wilcoxon signed-rank statistic for individual item scores. 

Change scores (∆) were calculated (T1–T2) for PAQ-C score and all hypothesised predictors. The first stage of analysis (Stage 1) involved regression modelling of ∆ PAQ-C score and ∆ for each predictor individually, including the interaction of ∆ predictor with cohort (Transition vs. Secondary school). This allows identification of predictors of the ∆ PAQ-C score that are common to, and differ between, young females who transition into secondary schooling and those who remain in secondary schooling across the same 12-month period. In Stage 2, the sample was stratified by cohort, and multiple regression modelling was conducted to identify independent predictors of ∆ PAQ-C separately in the Transition and Secondary school cohorts. Only variables found to be significant predictors of ∆ PAQ-C score in each cohort from Stage 1 modelling were entered into Stage 2 models. Separate Stage 2 models for psychological and social models were constructed where required. Multicollinearity among predictor variables was tested, using a cut-off for variance inflation factor (VIF) of 10; no risk to modelling from multicollinearity was identified. All models were adjusted for the baseline PAQ-C score (T1 PAQ-C). Initial correlation analysis revealed no association of pubertal status with change in any scores and, therefore, pubertal status was not included as a covariate in modelling. Multi-level analysis indicated a small and non-significant variance between schools (intra-class correlation coefficient; ICC) for PAQ-C score (ICC = 0.16 and −0.05 at time points 1 and 2, respectively). Nonetheless, Time 1 school was retained as a cluster variable in all models to provide conservative parameter estimates accounting for clustering within schools. Analyses were carried out using Stata 11.0 (Statacorp, College Station, TX, USA). The Department of Education and Children’s Services (Protocol number DECS CS/11/104.3) and the University of South Australia Human Research Ethics Committee (Protocol number 0000023900) provided ethical approval.

## 3. Results

Females (*n* = 191) in socioeconomically diverse schools in Adelaide, South Australia, volunteered to participate. This represents a response rate of 38% of girls who were invited from participating schools. Each girl was surveyed at two time points (T1 and T2), 12 months apart; 99 girls started in the final year of elementary school at T1 (Transition cohort) with 61 (62%) retained at T2; and 92 started in the first year of secondary school at T1 (Secondary cohort) with 57 (62%) retained at T2. In the Transition cohort, those retained were no different to those who dropped out at T2 in PAQ-C (*p* = 0.42), while those who dropped out were younger (*p* = 0.0007). In the Secondary cohort, those retained were no different to those who dropped out at T2 in PAQ-C *(p* = 0.75), while those who dropped out were also younger than those retained at T2 (*p* = 0.005). 

### Results of Statistical Modelling

#### Changes in Outcome and Predictor Variables Between Time Points, Separately by Cohort

As seen in Table 1, overall physical activity, as represented by the PAQ C score, declined in the Transition but not Secondary school cohort, between time points. The individual PAQ-C components that declined between time points in both cohorts were Lunch/recess play and physical activity on weekends. Among predictor variables, in the Transition cohort barriers self-efficacy and ‘parents play with’ both increased, while ‘friend play with’ decreased between time points. In the Secondary school cohort, ‘Parents play with’ increased, while the presence of bullies in the playground decreased between time points.

Table 2 displays the results of Stage 1 regression modelling of the change in PAQ-C score (∆ PAQ-C) where each predictor (change score) was entered individually. All significant associations were in the expected directions. In the psychological domain, ∆ Good at, ∆ Barriers self-efficacy, and ∆ Like PA predicted ∆ PAQ-C in the whole sample (see Table 2). The interaction term (Transition vs. Secondary school) was significant for ∆ Perceived outcomes and ∆ Barriers self-efficacy; stratified modelling revealed that these variables were associated with ∆ PAQ-C in the Secondary school cohort only (see Table 2). In the social domain, ∆ Friend encourage and ∆ Friend play with both predicted ∆ PAQ-C in the whole sample (see Table 2). The interaction term (Transition vs. Secondary school) was significant for ∆ Friend encourage; stratified modelling revealed that ∆ Friend encourage predicted ∆ PAQ-C in the Secondary school cohort only (see Table 2). 

Significant predictors of ∆ PAQ-C emerging from Stage 1 modelling were tested for independence in multiple regression models, stratified by Psychological and Social domains (see Table 3). Therefore, Stage 2 modelling tested independence among the following sets of predictors of ∆ PAQ-C; two psychological and one social variable in the Transition cohort; and four psychological and two social variables in the Secondary school cohort. In the psychological domain, ∆ Like PA was an independent predictor of ∆ PAQ-C in both cohorts. In the Secondary school cohort, ∆ Outcomes was also retained as an independent predictor of ∆ PAQ-C. In the social domain, ∆ Friends encourage was retained in the model of ∆ PAQ-C in the Secondary school cohort only. 

## 4. Discussion

This study examined associations of changes in self-reported physical activity and psychosocial correlates of physical activity over 12 months in two cohorts of adolescent girls, i.e., a Transition cohort who moved from elementary to secondary school, and a Secondary school cohort who remained in the same school. Overall, physical activity declined in the transition cohort but not the secondary cohort. This finding is consistent with other studies conducted in Australia [9] and internationally [13,15] where change of school was also associated with a greater reduction in physical activity levels. A change in school appears to have an impact on behaviour that is distinct from that associated with a within-school change in grade level. Notably, both our study and that of Marks and colleagues [9] reported a more significant decline in recess and lunch activity in the transitioning students, highlighting the importance of school break times for the promotion of physical activity in adolescent girls. The observed differences in school break time is significant as research has found physical activity accumulated during recess and lunch periods contributes a small, yet significant, amount to adolescents’ overall daily physical activity levels [10].

These results suggest that secondary schools should be cognisant of the physical activity opportunities provided for adolescents during break times. While school breaks are a time when students should maintain discretionary choice over their activity, structured opportunities and encouragement for physically active options can influence physical activity levels [25]. The UK-based SPEEDY study found that students who moved from a primary school that did not provide extra-curricular physical activity opportunities at break times to a secondary school that did provide these opportunities had both smaller reductions in MVPA time and smaller increases in sedentary time compared to other students [15]. Break-time activity preferences of girls should be considered as they often differ from boys [26]. Qualitative research providing information on girls’ perceptions and attitudes has found recess time has a highly social function for girls. Younger girls (aged 10 years) reported preferences for smaller ‘cosy’ areas with equipment such as swings and climbing walls to ‘hang out’ in and expressed that they enjoyed moving in smaller groups during recess instead of just sitting [26].

The social function of school break times is also worthy of specific consideration during the transition and early years of secondary schooling. The transition cohort reported a decline in the ‘friend play with’ item likely due to a change in peer group composition and social norms after moving to secondary school [14]. The effect of friend support on physical activity is likely to extend beyond school break times to overall physical activity as evidenced by the friend support item independently predicting physical activity change in the secondary cohort. An American study found that a physical activity decline over a three year period, which encompassed a move from elementary to middle school (akin to the move from primary to secondary school in this study), was least pronounced in those students who perceived more parent and friend support compared to those who reported the largest decrease in friend support [13]. The few physical activity interventions that have specifically targeted the peer environment had little or no success in influencing daily physical activity levels [27,28,29]. Arguably, cross-sectional associations reported in much of the literature may be bi-directional, in that active adolescents may associate socially with like-minded and, therefore, supportive peers. Nevertheless, peer support is consistently found to influence adolescent physical activity [16] and should continue to be explored as an intervention strategy, particularly during the secondary school transition period.

The analyses of predictors of change in physical activity behaviour provide some indicators of other key psychosocial domains that are influential in adolescence. While physical activity behaviour correlates previously established in the literature of perceived outcomes, perceived barriers, and enjoyment (i.e., the ‘like PA’ item in the current study) of physical activity [12,16] were all significant predictors of change in univariate regression models in the current study, the only independent predictors of physical activity change in multivariate regression models were change in enjoyment in the transition cohort and changes in enjoyment, perceived outcomes, and friend encouragement in the secondary cohort. Enjoyment is an established mediator of physical activity behaviour change in children and adolescents [30]; however, how can schools influence enjoyment of physical activity, particularly during the early years of secondary schooling? Evidence-based strategies known to enhance enjoyment of physical activity in physical education and other organised physical activity settings include provision of choice, variety in activities, and the use of motivational music where appropriate [31]. Children also report enjoying physical activity experiences most when they feel socially connected to their peers and teachers [31], providing additional support for secondary school transition support programs to focus on social relations.

The strength of this study was the cohort design allowing the comparison of one-year changes in physical activity behaviour and correlates between girls who moved schools from primary to secondary school and girls who moved to a higher year level within the same school. A number of study limitations are worth noting. While a validated self-report instrument was used, issues of recall and subjective biases might have influenced the validity of the physical activity scores. However, as this study was also interested in perceptions, a change in the adolescent girls’ perception of their physical activity levels, regardless of the accuracy of that perception, is also relevant. The physical activity measure did not capture transport to school, which has previously been found to decline when students move to a secondary school campus [9]. There also may be an undetected participation bias as we were unable to collect any demographic or behavioural data from the girls who did not consent to participate.

## 5. Conclusions

The social-ecological framework highlights the complex interactions among psychological, social, environmental, and policy levels of influence on regular participation in physical activity. As young people transition to a higher level of schooling via a change in school, they enter a markedly different physical environment within which their behaviour is shaped by sociocultural norms, curriculum, and policies unique to the secondary school context. Unsurprisingly, enablers and barriers of physical activity differ between school levels, underscoring the challenge for physical activity promotion to better understand these differences to more effectively tailor strategies and direct limited allocated funding. The current study particularly highlighted school break times as settings within which physical activity of adolescent females declines precipitously within the transition phase, and therefore, represent a prime target for intervention efforts.

## Figures and Tables

**Table 1:** Changes in physical activity and predictor variables between time point 1 and time point 2 (12 months later).

Variables	Transition Cohort (*n* = 104)			Secondary School Cohort (*n* = 65)		
	Time 1	Time 2	*p* Value for Change	Time 1	Time 2	*p* Value for Change
Physical activity						
* PAQ-C score	2.90 (0.73)	2.45 (0.78)	**<0.0001**	2.46 (0.71)	2.34 (0.61)	0.14
^#^ PE	4 (3.5, 4)	4 (1, 5)	0.12	4 (1, 4)	4 (2, 5)	0.35
^#^ Lunch/recess	3 (2, 4)	2 (2, 2)	**<0.0001**	2 (2, 2)	2 (1, 2)	**0.0003**
^#^ After school	3 (2, 4)	3 (1, 3)	0.07	3 (2, 3)	3 92, 3)	0.95
^#^ evenings	2 (1, 3)	2 (1, 3)	0.10	2 (1, 3)	2, (1, 3)	0.21
^#^ weekends	3 (2, 3)	2 (2, 3)	0.03	3 (2, 3)	2 91, 3)	**0.008**
Predictors						
*Psychological*						
* Perceived Outcomes	1.82 (0.51)	1.91 (0.54)	0.88	1.88 (0.48)	1.86 (0.48)	0.38
* Barriers self-efficacy	2.07 (0.57)	2.34 (0.68)	**0.008**	2.31 (0.58)	2.48 (0.71)	0.12
^#^ Good at	2 (2,3)	2 (2,3)	0.32	2 (2, 3)	2 (2, 3)	0.27
^#^ Don’t like PA feel	3 (2, 4)	3 (3, 4)	0.52	3 (2.5, 4)	4 (3, 4)	0.10
*Social*						
* Father support	3.68 (1.12)	3.44 (1.15)	0.19	3.36 (1.19)	3.43 (1.26)	0.69
* Mother support	3.94 (1.04)	3.91 (1.09)	0.85	3.64 (0.95)	3.55 (1.13)	0.59
* Parent play with	3.14 (1.03)	3.48 (0.97)	0.04	3.17 (1.15)	3.75 (1.03)	**0.002**
^#^ Right clothing	2 (1, 2)	1 (1, 2)	0.45	2 (1, 2)	2 (1, 2)	0.82
^#^ TV rules	2.25 (2, 3)	2.25 (1.75, 2.5)	0.66	2 (1.75, 2.5)	2.25 (1.15, 2.75)	0.06
^#^ Friends encourage	4 (3, 5)	4 (3, 5)	0.06	3 (3, 4)	3 (3, 4)	0.66
^#^ Friend play with	5 (4, 6)	4 (3, 5)	**0.0001**	4 (3, 5)	4 (3, 5)	0.66
^#^ Kids tease	4 (3, 5)	4 (3, 5)	0.13	4 (3, 5)	4 (3, 5)	0.97
^#^ Bullies	5 (4, 5)	4 (4, 5)	0.16	4 (3, 5)	5 (4, 5)	**0.01**

Note: * Comparisons by paired t-test; ^#^ comparisons by Wilcoxon rank order. *p* values denoting significant differences are shown in bold font. PAQ-C = Physical Activity Questionnaire for Older Children; PE = physical education; PA = physical activity.

**Table 2:** Results of stage 1 regression modelling of change in PAQ-C by cohort.

	Whole Sample				Transition Cohort			Secondary School Cohort		
Predictor	Coefficient	Robust SE	*p* for Main Effect	*p* for Interaction	Coefficient	Robust SE	*p*	Coefficient	Robust SE	*p*
*Psychological*										
∆ Perceived Outcomes	0.137	0.078	0.095	0.001	0.039	0.082	0.642	0.055	0.088	**0.004**
∆ Barriers self-efficacy	0.140	0.061	**0.033**	0.014	0.104	0.076	0.194	0.335	0.094	**0.008**
∆ Good at	0.092	0.032	**0.010**	0.475						
∆ Like PA	0.141	0.027	**0.0001**	0.951						
∆ Don’t like feel	0.004	0.081	0.961	0.689						
*Social*										
∆ Father support	0.060	0.042	0.179	0.095						
∆ Mother support	0.030	0.560	0.591	0.994						
∆ Parent play with	0.020	0.036	0.576	0.550						
∆ Right clothing	0.073	0.048	0.140	0.124						
∆ TV rules	-0.020	0.068	0.770	0.666						
∆ Friend encourage	0.100	0.046	**0.041**	**<0.0001**	0.025	0.029	0.406	0.217	0.039	**<0.0001**
∆ Friend play with	0.181	0.052	**0.002**	0.973						
∆ Kids tease	0.036	0.023	0.132	0.723						
∆ Bullies	0.016	0.076	0.840	0.619						

Note: all models controlled for T1 PAQ-C score; PA = physical activity. *p* values denoting significant differences are shown in bold font.

**Table 3:** Results of stage 2 multiple regression modelling of change in PAQ-C by cohort.

Predictor	Coefficient	Robust SE	*p*
**Transition Cohort**			
Psychological			
∆ Like PA	0.139	0.039	**0.003**
			Model R^2^ = 0.088
**Secondary School Cohort**			
Psychological			
∆ Like PA	0.109	0.032	**0.010**
∆ Perceived Outcomes	0.303	0.119	**0.034**
			Model R^2^ = 0.290
Social			
∆ Friend encourage	0.217	0.039	**<0.0001**
			Model R^2^ = 0.340

Note: all models controlled for T1 PAQ-C score; PA = physical activity. *p* values denoting significant differences are shown in bold font.
